# Supplementary material for: Radixin modulates the function of outer hair cell stereocilia
Source: Commun Biol. 2020 Dec 23;3:792. doi: 10.1038/s42003-020-01506-y (PMC7758333; doi:10.1038/s42003-020-01506-y)
Supplement: Supplementary file 2 — Supplementary Information [file 42003_2020_1506_MOESM2_ESM.pdf]

Radixin modulates the function of outer hair cell stereocilia

Sonal Prasad[1*], Barbara Vona[2], Marta Diñeiro[3], María Costales[4], Rocío González-Aguado[5], Ana Fontalba[6], Clara Diego-Pérez[7], Asli Subasioglu[8], Guney Bademci[9], Mustafa Tekin[9, 10, 11], Rubén Cabanillas[12], Juan Cadiñanos[3], Anders Fridberger[1*]

[1]Department of Biomedical and Clinical Sciences, Linköping University, SE-581 83 Linköping, Sweden

[2] Department of Otorhinolaryngology, Head and Neck Surgery, Tübingen Hearing Research Centre, Eberhard Karls University Tübingen, 72076 Tübingen, Germany

[3] Laboratorio de Medicina Molecular, Instituto de Medicina Oncologica y Molecular de Asturias, 33193 Oviedo, Spain

[4] Department of Otorhinolaryngology, Hospital Universitario Central de Asturias, 33011 Oviedo, Spain

[5] Department of Otorhinolaryngology, Hospital Universitario Marqués de Valdecilla, 39008 Santander, Spain

[6] Department of Genetics, Hospital Universitario Marqués de Valdecilla, 39008 Santander, Spain

[7] Department of Otorhinolaryngology, Hospital Universitario de Salamanca, 33007 Salamanca, Spain

[8] Department of Medical Genetics, Izmir Ataturk Education and Research Hospital, Izmir 35360, Turkey

[9] John P. Hussman Institute for Human Genomics, University of Miami Miller School of Medicine, Miami, FL 33136, USA

[10] Department of Otolaryngology, University of Miami Miller School of Medicine, Miami, FL 33136, USA

[11] Dr. John T. Macdonald Department of Human Genetics, University of Miami Miller School of Medicine, Miami, FL 33136, USA

[12] Área de Medicina de Precisión, Instituto de Medicina Oncologica y Molecular de Asturias, 33193 Oviedo, Spain

*Corresponding authors: S.P. (sonal.prasad@liu.se) and A.F. (anders.fridberger@liu.se)

Supplementary figures 1-3

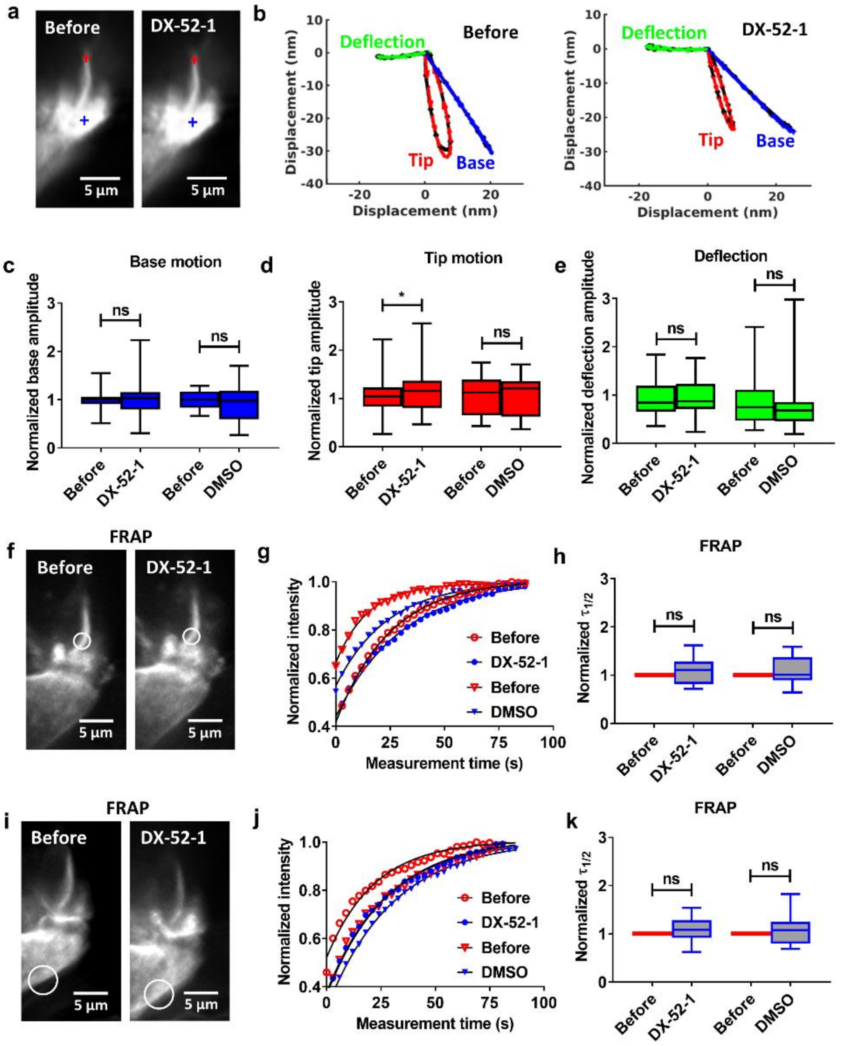

Supplementary Figure 1. DX-52-1 induced effects on the IHC stereocilia functions.

a Time-resolved confocal images acquired during sound stimulation showing the morphology of the inner hair cell stereocilia bundle is intact before and after DX-52-1 injection. **b** No significant change in sound-evoked motion of the bundle tip (red) and base (blue) before (left) and after (right) DX-52-1 injection observed along with the absence of change in deflection (green) in an example preparation. The stimulus was a pure tone at 220 Hz and 80 dB sound pressure level. **c - e** Averaged bundle motion change at the base of inner hair cell stereocilia (blue bar), at their tip (red bar) and the deflection of the bundle (green bar). Data were normalized to the base trajectory amplitude recorded before the injection. Averaged data from DX-52-1 (n=18) and DMSO (n=8) individual preparations ± s.d. **f** FRAP experiment showing no change in the stereocilia bundle morphology before and after DX-52-1 injection, except for slight change in the dye intensity. **g** Normalized traces of the fluorescence intensity showing no change in the membrane dynamics during the fluorescence recovery in the bundle region of interest before and after DX-52-1 and DMSO injection in an example preparation. **h** Fitting the experimental data to single phase exponential fit model showed a non-significantly slower recovery of bundle fluorescence with increased $\tau_{1/2}$ after DX-52-1 injection (n=20) and with no change in the diffusion time after DMSO injection (n=12). Data are the means ± s.d. **i** No change in the cell somatic membrane morphology before and after DX-52-1 injection. **j** Normalized traces of the fluorescence intensity showing no change in the membrane dynamics during the fluorescence recovery before and after DX-52-1 and DMSO injection in an example preparation. **k** Fitting the experimental data to single phase exponential fit model showed a non-significantly slower recovery of cell membrane fluorescence with increased $\tau_{1/2}$ after DX-52-1 injection (n=20) and with no change in the diffusion time after DMSO injection (n=12). Data are the means ± s.d. All data sets were normalized to the data recorded before injection. *P<0.05; n.s., not significant; two-tailed paired t test.

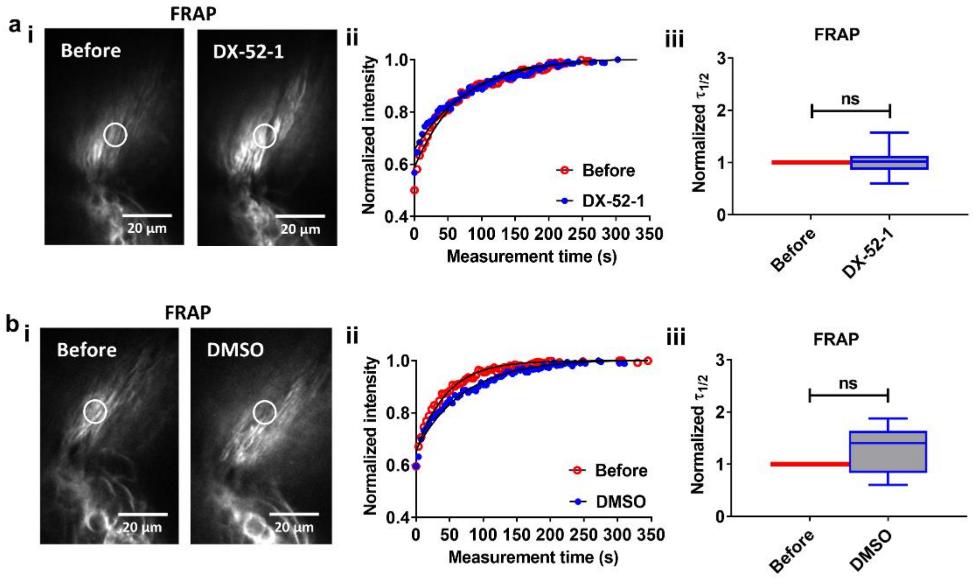

Supplementary Figure 2. DX-52-1 induced effects on the auditory nerve fibres.

(a, b) i FRAP experiment showing no change in the morphology of nerve fibres before and after DX-52-1 and DMSO injection, except for slight change in the dye intensity. **ii** Normalized traces of the fluorescence intensity showing no change in the membrane dynamics during the fluorescence recovery in the neuronal region of interest measuring the diffusion time of the dye before and after DX-52-1 and DMSO injection in an example preparation. **iii** Fitting the experimental data to single phase exponential fit model showed a non-significantly slower recovery of auditory neurons fluorescence with increased $\tau_{1/2}$ after DX-52-1 injection (n=18) and after DMSO injection (n=8). Data are the means ± s.d. All data sets were normalized to the data recorded before injection. *P<0.05; n.s., not significant, two-tailed paired t test.

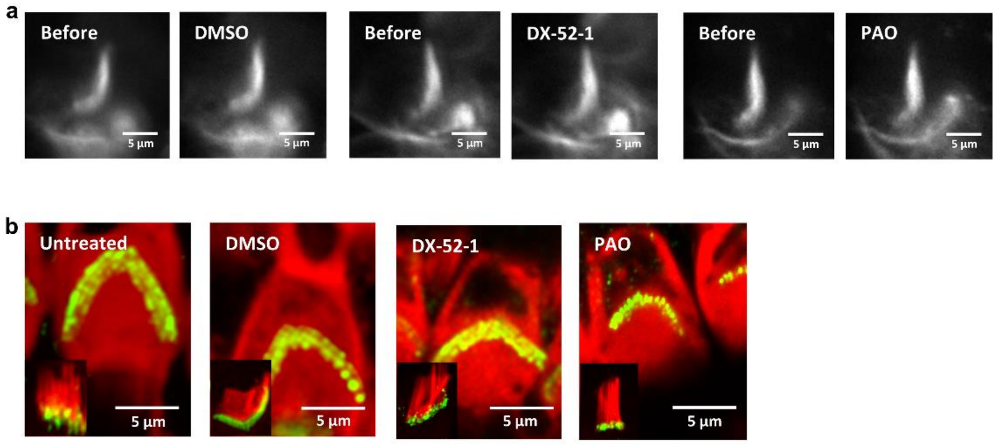

Supplementary Figure 3. Drug induced effect on the OHC stereocilia morphology.

a Confocal image of outer hair cell stereocilia showing no defects in the stereocilia morphology before and after DMSO, DX-52-1 and PAO injection. **b** Representative overlay confocal immunofluorescence images of sections of the organ of Corti labelled with a radixin-specific monoclonal antibody (green) as well as phalloidin (red, staining actin) upon treatment with DMSO, DX-52-1 and PAO. The hair bundles of the first-row outer hair cells are intensely labeled by the radixin antibody and the hair cell body labeled by actin showing intact hair bundle morphology with and without any drug treatment. Images were taken from the surface preparations of the apical turn. Inset showing a higher magnification view on the outer hair cell area showing predominance of radixin labeling near the stereocilia base and consistent actin labeling in the hair bundles.
