# Supplementary material for: Radixin modulates the function of outer hair cell stereocilia
Source: Commun Biol. 2020 Dec 23;3:792. doi: 10.1038/s42003-020-01506-y (PMC7758333; doi:10.1038/s42003-020-01506-y)
Supplement: Supplementary file 3 — Description of Additional Supplementary Files [file 42003_2020_1506_MOESM3_ESM.pdf]

Description of Additional Supplementary Files

File Name: Supplementary Data 1

Description: Source data underlying the graphs presented in the main body text figures and in the supplementary information figures.

File Name: Supplementary Data 2

Description: Information on variants. The variants are submitted to a clinical variant respository, ClinVar. The database includes variants and information about pathogenicity.
